# Anesthesia from the Local Application of Chloroform

**Published:** 1849-11

**Authors:** 


*Anesthesia from the Local Application of Chloroform.*—Mr. Higgenson brought forward the case of a lady, aged 25 years, in labor with her first child: the perineum had long been on the stretch by the head, which was tumefied by the pressure; the pain was great with each uterine contraction, but was referred entirely to the perineum, no pain being apparently felt from the uterine contraction itself.

About half a drachm of chloroform was poured upon a handkerchief in the ordinary manner, but instead of being applied to the mouth it was held in almost immediate contact with the perineum. The pain immediately ceased though the uterine contractions continued in full force; and the first intimation the patient had of the progress of the labor, was hearing the child cry. Her mind was not at all affected, nor was intellectual consciousness in any degree diminished.

He had observed the same thing, though in a less degree, when the chloroform had been applied to the sacrum in another case.

He had also applied this agent to the os uteri of a patient suffering from very severe dysmenorrhea, by means of a sponge placed in a curved glass speculum, which was introduced into the vagina. The pain almost immediately abated, and on its return, after some hours, the patient re-applied it herself with similar benefit.

Dr. Watson mentioned some cases confirmatory of its good effects when locally applied. He had painted it over a swelled testicle, with speedy relief to the pain, and had applied it along the course of the spine with a similar result in a case of acute spinal tenderness, which had not been relieved by other treatment. He had also applied it to the surface of a large mammary abscess prior to opening it, which was afterwards done without suffering to the patient; and also to the vulva of a woman before cauterizing the orifice of the urethra. It had relieved the cramp and collapse in a case of English cholera when laid upon the epigastrium, and had abated the pain almost immediately when painted around the edge of a surface to which potassa fusa had been applied for the purpose of forming an issue.—*Lon. Med. Gaz.*

